# Supplementary material for: Spin liquid mediated RKKY interaction
Source: Sci Rep. 2019 Nov 27;9:17697. doi: 10.1038/s41598-019-53842-7 (PMC6881398; doi:10.1038/s41598-019-53842-7)
Supplement: Supplementary file 1 — Supplementary information [file 41598_2019_53842_MOESM1_ESM.pdf]

Spin liquid mediated RKKY interaction - Supplementary information

Henry F. Legg[1,*] **and Bernd Braunecker**[2]

[1]Institut für Theoretische Physik, Universität zu Köln, D-50937 Cologne, Germany
[2]SUPA, School of Physics and Astronomy, University of St Andrews, North Haugh, St Andrews, Fife KY16 9SS, United Kingdom
[*]hlegg@uni-koeln.de

1 Kondo-Yamaji decoupling of KHAF electron spin susceptibility

A second-order Green's function decoupling introduced by Kondo and Yamaji[1] can be used to calculate the key features of the spin susceptibility of the $S = 1/2$ KAFM required for our analysis. This has previously been utilised to calculate the specific heat and structure factor of the KAFM[2,3] and we follow those previous calculations closely here.

A first order decoupling of the spin Green's function - commonly known as a Tyablikov decoupling[4] - can be achieved by expanding in terms of expectation values of spin operators. We begin with the equation of motion for the dynamic spin susceptibility

$$\begin{aligned}\omega\chi_{ij}(\omega) &= \omega\langle\langle S_i^+;S_j^-\rangle\rangle_\omega = \langle[S_i^+,S_j^-]\rangle_{\omega=0} + \langle\langle[S_i^+,H];S_j^-\rangle\rangle_\omega \\ &= 2\delta_{ij}\langle S_i^z\rangle_0 + \sum_k J_{ik}\langle\langle S_i^zS_k^+ - S_i^+S_k^z;S_j^-\rangle\rangle_\omega,\end{aligned} \tag{1}$$

where in the second line we have inserted the Heisenberg Hamiltonian H_{sl} in the main text and $\langle\langle A;B\rangle\rangle_\omega$ is the time Fourier transform of the retarded Green's function of A and B,

$$\langle\langle A;B\rangle\rangle_t = -i\theta(t)\langle[A(t),B(0)]\rangle. \tag{2}$$

In a spin liquid there is no long-ranged order and as such $\langle S_i^x\rangle_0 = \langle S_i^y\rangle_0 = \langle S_i^z\rangle_0 = 0$ this means that a standard first order Tyablikov decoupling of the form

$$\langle\langle S_i^zS_k^+;S_j^-\rangle\rangle_\omega \to \langle S_i^z\rangle_0\langle\langle S_k^+;S_j^-\rangle\rangle_\omega, \tag{3}$$

will not allow us to calculate the spin-susceptibility from a self-consistent identity.

Instead we must decouple the susceptibility at second order by applying a Tyablikov-like decoupling to the equation of motion for the three point Green's function that appear in the spin susceptibility's equation of motion

$$\omega\chi_{ij}(\omega) = \omega\langle\langle S_k^+;S_j^-\rangle\rangle_\omega = \sum_k J_{ik}\left[\langle\langle S_i^zS_k^+;S_j^-\rangle\rangle_\omega - \langle\langle S_i^+S_k^z;S_j^-\rangle\rangle_\omega\right], \tag{4}$$

Where the equation of motion for the 3-point Green's functions can be written

$$\begin{aligned}&\omega\langle\langle S_i^+S_k^z;S_j^-\rangle\rangle_\omega = \langle[S_i^+S_k^z,S_j^-]\rangle_0 + \langle\langle[S_i^+S_k^z,H];S_j^-\rangle\rangle_\omega \\ &= \langle[S_i^+S_k^z,S_j^-]\rangle_0 + \langle\langle S_i^+[S_k^z,H] + [S_i^+,H]S_k^z;S_j^-\rangle\rangle_\omega \\ &= (\delta_{i,j}-\delta_{k,j})\langle S_i^+S_k^-\rangle_0 + \frac{1}{2}\sum_n J_{kn}\langle\langle S_i^+(S_k^+S_n^- - S_k^-S_n^+);S_j^-\rangle\rangle_\omega + \sum_n J_{in}\langle\langle(S_n^+S_i^z - S_i^+S_n^z)S_k^z;S_j^-\rangle\rangle_\omega.\end{aligned} \tag{5}$$

There are two types of three operator Green's functions that appear in Eq. (5) these have the form $\langle\langle S_i^+S_k^-S_n^+;S_j^-\rangle\rangle$ and $\langle\langle S_i^+S_n^zS_k^z;S_j^-\rangle\rangle$. A Kondo-Yamaji coupling expands these operators in terms of finite spin-spin correlations such as $\langle S_i^+S_j^-\rangle_0$ and $\langle S_i^zS_j^z\rangle_0$,

$$\langle\langle S_i^+S_k^-S_n^+;S_j^-\rangle\rangle \to \alpha\langle S_i^+S_k^-\rangle_0\langle\langle S_n^+;S_j^-\rangle\rangle_\omega + \alpha\langle S_k^-S_n^+\rangle_0\langle\langle S_i^+;S_j^-\rangle\rangle_\omega \tag{6}$$

and

$$\langle\langle S_i^+ S_n^z S_k^z ; S_j^- \rangle\rangle_\omega \to \alpha \langle S_n^z S_k^z \rangle_0 \langle\langle S_i^+ ; S_j^- \rangle\rangle_\omega, \tag{7}$$

where α is found self-consistently, as specified below. Inserting these decoupled equation of motion for the three-point functions $\langle\langle S_i^z S_k^+ ; S_j^- \rangle\rangle_\omega$ and $\langle\langle S_i^+ S_k^z ; S_j^- \rangle\rangle_\omega$ gives an equation entirely in terms of $\langle\langle S_k^+ ; S_j^- \rangle\rangle_\omega$, self-consistent parameters, and exchange matrices of the Heisenberg model such that

$$\begin{aligned} \omega^2 \langle\langle S_k^+ ; S_j^- \rangle\rangle_\omega = {} & 2c_1 J_{ij} - 8J\delta_{ij} c_1 - (c + 2\alpha c_1) J \sum_k J_{ik} \langle\langle S_k^+ ; S_j^- \rangle\rangle_\omega \\ & + 4cJ^2 \langle\langle S_k^+ ; S_j^- \rangle\rangle_\omega + \alpha c_1 J \sum_k J'_{ik} \langle\langle S_k^+ ; S_j^- \rangle\rangle_\omega \end{aligned} \tag{8}$$

where (as in Bernhard *et al.*[2])

$$\langle S_i^+ S_j^- \rangle = \begin{cases} c_1, & i,j \text{ nearest neighbors (NN)}, \\ c_2, & i,j \text{ next-NN (shortest distance)}, \\ c_2', & i,j \text{ next-NN (longest distance)}, \end{cases} \tag{9}$$

and J'_{ij} is defined by

$$J'_{ij} = \begin{cases} J > 0, & \text{if } i \text{ and } j \text{ are next-nearest neighbors}, \\ 0, & \text{otherwise}. \end{cases} \tag{10}$$

Replacing $\chi(i,j,\omega) = \langle\langle S_k^+ ; S_j^- \rangle\rangle_\omega$ and taking the Fourier transform with respect to the triangular Bravais lattice gives a matrix equation of the form

$$\sum_b M^{ab}(\mathbf{q},\omega) \chi^{bc}(\mathbf{q},\omega) = N^{ac}(\mathbf{q},\omega), \tag{11}$$

where we have defined the matrices

$$M^{ab}(\mathbf{q},\omega) = (\omega^2 - 4cJ^2)\delta_{ab} + J(c + 2\alpha c_1) J_{ab}(\mathbf{q}) - \alpha c_1 J J'_{ab}(\mathbf{q}) \tag{12}$$

and

$$N^{ab}(\mathbf{q},\omega) = 2c_1 J_{ab}(\mathbf{q}) - 4Jc_1 \delta_{ab}, \tag{13}$$

with $J_{ab}(\mathbf{q})$ and $J'_{ab}(\mathbf{q})$ the Fourier transforms of the exchange matrices J_{ij} and J'_{ij} of the Heisenberg model on kagome[2] for crystal momentum $\boldsymbol{q}$ and intra-unit cell positions a,b. These Fourier transformed matrices read

$$J_{ab}(\mathbf{q}) = 2J\cos(\mathbf{q}\cdot\mathbf{r}_{ab}) - \delta_{ab}, \tag{14}$$

where $\mathbf{r}_{ab}$ are the real space lattice vectors between neighboring sites a and b, and

$$J'_{ab}(\mathbf{q}) = J \sum_{\mathbf{r}'_{ab}} \cos(\mathbf{q}\cdot\mathbf{r}'_{ab}), \tag{15}$$

with $\mathbf{r}'_{ab}$ the four real space lattice vectors between next-nearest-neighboring sites (by lattice distance) a and b. The full susceptibility matrix $\chi^{ab}(\mathbf{q})$ therefore includes terms that couple sites on different plaquettes and in particular on the same sublattice, such terms corresponding to the term proportional to c_2' from the longer distance next-nearest neighbours and appear on the diagonal of the susceptibility matrix. The presence of these next-nearest neighbour terms encourages orderings between neighbouring plaquettes.

The eigenvalues of the susceptibility matrix are shown in the main text and the qualitative features only weakly depend on the values of the decoupling parameters c_1, c_2, and c_2'. In particular, due to the frustrated nature of the kagome lattice both $J_{ab}(\mathbf{q})$ and $J'_{ab}(\mathbf{q})$ have an eigenvalue which is independent of $\mathbf{q}$ and shares a common eigenspace,[5] as a direct consequence of only these two matrices appearing in the equation for the suscepibility matrix $\chi^{ab}(\mathbf{q},\omega=0)$, it also has a flat band.

2 Ground state energy lower bound

In the main text we motivate the use of a Luttinger-Tisza expansion of the spins reading, $\mathbf{I}_a(\mathbf{q}) = \sum_\nu \mathbf{W}_\nu(\mathbf{q}) U_{a,\nu}(\mathbf{q})$, where the $\mathbf{W}_\nu(\mathbf{q})$ are (orthogonal) directions in spin-space. This allows us to find the minimum possible energy of the slRKKY interaction subject on average spin-length within the unit cell, which is also know as the "weak constraint" on spin-length.

Inserting this Luttinger-Tisza expansion of spins in terms of the eigenvectors of the susceptibility matrix $\chi^{ab}(\mathbf{q})$ into the slRKKY Hamiltonian gives

$$H_{\text{eff}} = \frac{A^2}{4J} \sum_{\mathbf{q},a,b,\nu,\nu'} \chi^{ab}(\mathbf{q}, \omega = 0)\, U_{\mathbf{q}}^{a,\nu} U_{-\mathbf{q}}^{b,\nu'} \mathbf{W}^\nu \cdot \mathbf{W}^{\nu'}. \tag{16}$$

Using the fact that $U_{\mathbf{q}}^{a,\nu}$ is an eigenvector of the static susceptibility matrix with eigenvalue $\lambda^\nu(\mathbf{q})$ gives

$$H_{\text{eff}} = \frac{A^2}{4J} \sum_{\mathbf{q},b,\nu,\nu'} \lambda^\nu(\mathbf{q}) U_{\mathbf{q}}^{b,\nu} U_{-\mathbf{q}}^{b,\nu'} \mathbf{W}^\nu \cdot \mathbf{W}^{\nu'}. \tag{17}$$

Because the eigenvectors $U_{\mathbf{q}}^{a,\nu}$ are orthonormal this then reduces to

$$H_{\text{eff}} = \frac{A^2}{4J} \sum_{\mathbf{q},\nu} \lambda^\nu(\mathbf{q}) \mathbf{W}^\nu \cdot \mathbf{W}^\nu. \tag{18}$$

The weak constraint on average spin length in Fourier space reads $\sum_{\mathbf{q},a} I_{\mathbf{q}}^a \cdot I_{-\mathbf{q}}^a = NI^2$ means that we require $\mathbf{W}^\nu \cdot \mathbf{W}^\nu = I^2$ and so the minimum ground state energy with this weak constraint applied is

$$E_{\text{GS}} = \frac{A^2 N I^2 \lambda_{\text{min}}}{4J}. \tag{19}$$

It is important to note the key difference between the slRKKY interaction and nearest neighbour classical KAFM is that the former is extended and therefore sensitive to the alignment of the spins on the same sublattice because the susceptibility matrix includes diagonal elements coupling these spins, whereas the latter has fewer spin constraints and therefore a much larger ground state degeneracy.[6] In particular, for slRKKY on the KAFM, once the spins on one plaquette are fixed the entire nuclear magnetic order is itself fixed since the Luttinger-Tisza energy minimum can only be achieved by an ordering over the whole lattice.

3 Multiple-Q ordering

In addition to the minimum energy configurations discussed in the main text characterised bya single $\boldsymbol{Q}$ ordering vector, a state mixing different $\boldsymbol{Q}$ values is also possible. Due to the symmetry of the susceptibility and the fact that the $\boldsymbol{Q} = 0$ ordering is doubly degenerate the slRKKY energy is also minimised for the multiple-$\boldsymbol{Q}$ state:

$$\begin{pmatrix} \boldsymbol{I}_i^1 \\ \boldsymbol{I}_i^2 \\ \boldsymbol{I}_i^3 \end{pmatrix} = I \begin{pmatrix} 1 \\ -1/2 \\ -1/2 \end{pmatrix} \hat{\boldsymbol{z}} + I\frac{\sqrt{3}}{2} \Big(\cos(Q_y y)\hat{\boldsymbol{x}} + \sin(Q_y y)\hat{\boldsymbol{y}} \Big) \begin{pmatrix} 0 \\ 1 \\ -1 \end{pmatrix}. \tag{20}$$

Such an ordering is non-coplanar and contains to two $\boldsymbol{Q}$s, namely: $\boldsymbol{Q} = 0$ in the spin $\hat{\boldsymbol{z}}$ direction and $\boldsymbol{Q}_y = Q_y \hat{\boldsymbol{y}}$ in the $\hat{\boldsymbol{x}} - \hat{\boldsymbol{y}}$ plane, where the x, y, z directions in spin space can be chosen arbitrarily.

To see why such an state is allowed to exist it is easiest to write this multiple-$\boldsymbol{Q}$ state in Fourier space

$$\begin{pmatrix} \boldsymbol{I}_{\boldsymbol{q}}^1 \\ \boldsymbol{I}_{\boldsymbol{q}}^2 \\ \boldsymbol{I}_{\boldsymbol{q}}^3 \end{pmatrix} = IN_{\text{Br}} I \delta_{\boldsymbol{q},\boldsymbol{0}} \begin{pmatrix} 1 \\ -1/2 \\ -1/2 \end{pmatrix} \hat{\boldsymbol{z}} + IN_{\text{Br}} \frac{\sqrt{3}}{4} \Big(\delta_{\boldsymbol{q},\boldsymbol{Q}_y}(\hat{\boldsymbol{x}} - i\hat{\boldsymbol{y}}) + \delta_{\boldsymbol{q},-\boldsymbol{Q}_y}(\hat{\boldsymbol{x}} + i\hat{\boldsymbol{y}}) \Big) \begin{pmatrix} 0 \\ 1 \\ -1 \end{pmatrix}. \tag{21}$$

We see that this is a true ground state of the slRKKY interaction on kagome because: 1) The state satisfies the hard-constraint on spin-length, that is the constraint $|I_i| = I$ for each spin – in contrast to the weak spin constraint where spin length is only imposed on average; 2) $(1, -1/2, -1/2)^T$ is an eigenvector of the susceptibility at $\boldsymbol{Q} = 0$ with minimum eigenvalue; 3) $(0, 1, -1)^T$ is an eigenvector of the susceptibility at $\boldsymbol{Q} = Q_y \hat{\boldsymbol{y}}$ with minimum eigenvalue.

Point 3) is the key to understanding why such a state is allowed. The susceptibility of the KAFM is doubly degenerate at $\boldsymbol{Q} = 0$ with an eigen-space spanned by $(1, -1/2, -1/2)^T$ and $(0, 1, -1)^T$. This means we have the possibility to use two

Luttinger-Tisza vectors to make a ground state. However, by symmetry, the vectors $(0,1,-1)^T$, $(1,0,-1)^T$, and $(1,-1,0)^T$ which are in the minimum eigen-basis at $\boldsymbol{Q}=0$ are the eigenvectors along the direction $\pm\hat{\boldsymbol{y}}$, the direction $\pm\sqrt{3}/2\hat{\boldsymbol{x}}\mp 1/2\hat{\boldsymbol{y}}$, and the direction $\mp\sqrt{3}/2\hat{\boldsymbol{x}}\mp 1/2\hat{\boldsymbol{y}}$, respectively. This means that we can start with a pure $\boldsymbol{Q}=0$ state and then move the component corresponding to one of the vectors $(0,1,-1)^T$, $(1,0,-1)^T$, or $(1,-1,0)^T$ along $\boldsymbol{Q}$ parallel to the direction in which it remains a lowest eigenvector and the state will remain a ground state.

This multiple-$\boldsymbol{Q}$ state is therefore only possible due to a combination of features of the kagome lattice and the KAFM susceptibility: 1) One of the vectors at $\boldsymbol{Q}=0$ does not affect one of the spins in the unit cell, 2) the susceptibility at $\boldsymbol{Q}=0$ is degenerate, 3) $\boldsymbol{Q}=0$, on each sublattice, only uses up one of three available orthogonal directions in spin space, and 4) the symmetry of kagome means these vectors remain the minimum eigenvalue vector along the high symmetry directions listed above. Since $\sqrt{3}\times\sqrt{3}$ order does not satisfy 1, 2, or 3, $\boldsymbol{Q}=0$ order possess no further Luttinger-Tisza vectors, and there are no further $\boldsymbol{Q}$ vectors that minimise the slRKKY energy we see that it is likely the multi-Q state and symmetry partners are the only possible multiple-$\boldsymbol{Q}$ states of slRKKY.

4 Holstein-Primakoff

As outlined in the main text: We can chose a basis within the unit cell with respect to the direction $\hat{\boldsymbol{z}}$ defined by a central spin and with the other two spins defined within this basis as $\mathbf{I}_2'(\mathbf{Q}_n)=R(\theta_2)\mathbf{I}_2(\mathbf{Q}_n)$ and $\mathbf{I}_3'(\mathbf{Q}_n)=R(\theta_3)\mathbf{I}_3(\mathbf{Q}_n)$, where $R(\theta_i)$ is the rotation matrix about spin y-axis and θ_i is the angle between the first spin and the spin on site i.

Since the nuclear spins are large we can take a $1/I$ Holstein-Primakoff expansion to estimate the nuclear spin-wave spectrum,[7] which in Fourier space reads

$$\begin{aligned} I'^{z}_{a,\mathbf{Q}_n}(\mathbf{q}) &= N_{Br}\delta_{\mathbf{q}0}I-\sum_{\mathbf{p}}a^{\dagger a}_{\mathbf{p}}a^{a}_{\mathbf{p}+\mathbf{q}}, \\ I'^{x}_{a,\mathbf{Q}_n}(\mathbf{q}) &= \sqrt{\frac{N_{Br}I}{2}}(a^{a}_{\mathbf{q}}+a^{\dagger a}_{-\mathbf{q}}), \\ I'^{y}_{a,\mathbf{Q}_n}(\mathbf{q}) &= i\sqrt{\frac{N_{Br}I}{2}}(a^{\dagger a}_{-\mathbf{q}}-a^{a}_{\mathbf{q}}), \end{aligned} \tag{22}$$

where $a^{\dagger a}_{\mathbf{q}}, a^{a}_{\mathbf{q}}$ are the bosonic creation and annihilation operators, respectively, for magnons at momentum $\mathbf{q}$ on sublattice a about the mean field equilibrium ordering vector $\mathbf{Q}_n$.

Inserting the Holstein-Primakoff expansion into the slRKKY Hamiltonian, to zeroth-order in bosonic operators – order I^2 – gives E_{min} the minimal ground state energy

$$H^{(0)}=\frac{A^2}{4J}\sum_{\mathbf{q},a,b}\chi^{ab}(\mathbf{Q}_n,0)\cos(\theta_a-\theta_b)N_{Br}I^2=\frac{A^2NI^2\lambda_{\min}}{4J}=E_{\min}, \tag{23}$$

where the second equality follows from the fact that we chose the angles θ_a for our equilibrium ordering such that the matrix $NI^2\cos(\theta_a-\theta_b)=\mathbf{I}_a(\mathbf{Q}_n)\cdot\mathbf{I}_b(-\mathbf{Q}_n)$ outputs the minimum eigenvalue of $\chi^{ab}(\mathbf{Q}_n,0)$.

The next order of the expansion comes from mixed x and z spin components. These will equal zero if

$$\sum_b \chi^{ab}(\mathbf{Q}_n,0)\sin(\theta_a-\theta_b)=0. \tag{24}$$

The construction of $\cos(\theta_a-\theta_b)$ to minimise the energy of the slRKKY Hamiltonian means that Eq. (24) must hold or else one could rotate and achieve a lower minimum than that set by θ_a. Conversely the the condition at the minima that the exchange interaction has equal magnitude on each site and the above condition in Eq. (24) - that the effective magnetic field on each site vanishes – set limits on the possible regular periodic orderings we could find. Given these two conditions the mutual angles between the three spins in the Fourier-space unit cell – that is, before translation to real space – must either be collinear, which automatically satisfies Eq. (24), or have angles of 60° or 120° between them such that the terms from the susceptibility matrix cancel. As such the only orderings that can satisfy both conditions are those orderings found in the main text.

In the presence of a magnetic field, unlike in the free field case, the linear terms no longer cancel because the additional Zeeman term – after rotation on each site, as above – contains components of $I_2'^x$, $I_3'^x$ from the spins that are not aligned with the field. This causes linear terms in the spin-wave operators,

$$H^{(1)}=\frac{\sqrt{3}g_{\text{nuc}}N_{Br}IB}{4}(-a^2_{\mathbf{q}}-a^2_{-\mathbf{q}}+a^3_{\mathbf{q}}+a^3_{-\mathbf{q}}). \tag{25}$$

These terms represent the fact that the effective magnetic field from the RKKY and the external field now compete and the nuclear spins will cant upwards slightly from the true 120° from the spin parallel to the field, further increasing magnetic field would eventually form a ferromagnetic arrangement. For our purposes we assume that fields are sufficiently small that the order can be approximated by the original mean field RKKY result.

5 Spin-wave diagonalisation

We wish to solve the general problem of finding the transformation of N species of bosons that diagonalises a block-diagonal spin-wave Hamiltonian in terms of new operators $\mathbf{d}(\mathbf{q})$ and $\mathbf{d}^\dagger(\mathbf{q})$ to a Hamiltonian of the form

$$\begin{aligned} H &= \sum_{\mathbf{q}} (\mathbf{a}^\dagger_{-\mathbf{q}}, \mathbf{a}_{\mathbf{q}}) \begin{pmatrix} \mathbf{\Gamma} & \mathbf{\Lambda} \\ \mathbf{\Lambda} & \mathbf{\Gamma} \end{pmatrix} \begin{pmatrix} \mathbf{a}_{-\mathbf{q}} \\ \mathbf{a}^\dagger_{\mathbf{q}} \end{pmatrix} \\ &= \sum_{\mathbf{q}} (\mathbf{d}^\dagger_{-\mathbf{q}}, \mathbf{d}_{\mathbf{q}}) \begin{pmatrix} \mathbf{\Delta}(\mathbf{q}) & 0 \\ 0 & \mathbf{\Delta}(\mathbf{q}) \end{pmatrix} \begin{pmatrix} \mathbf{d}_{-\mathbf{q}} \\ \mathbf{d}^\dagger_{\mathbf{q}} \end{pmatrix}, \end{aligned} \tag{26}$$

where $[\mathbf{\Delta}]_{ab} = \omega_a \delta_{ab}$ is a diagonal matrix of the spin wave spectra and the transformation matrix $\mathbf{D}$ between operators

$$\begin{pmatrix} \mathbf{d}_{-\mathbf{q}} \\ \mathbf{d}^\dagger_{\mathbf{q}} \end{pmatrix} = \mathbf{D} \begin{pmatrix} \mathbf{a}_{-\mathbf{q}} \\ \mathbf{a}^\dagger_{\mathbf{q}} \end{pmatrix} = \begin{pmatrix} \mathbf{u} & \mathbf{v} \\ \mathbf{v} & \mathbf{u} \end{pmatrix} \begin{pmatrix} \mathbf{a}_{-\mathbf{q}} \\ \mathbf{a}^\dagger_{\mathbf{q}} \end{pmatrix}, \tag{27}$$

with $N \times N$ matrices $\mathbf{u}$ and $\mathbf{v}$. To preserve bosonic commutation relations these must satisfy

$$\mathbf{u}\mathbf{u}^T - \mathbf{v}\mathbf{v}^T = \mathbb{1}_N \tag{28}$$

and

$$\mathbf{u}\mathbf{v}^T = \mathbf{v}\mathbf{u}^T, \tag{29}$$

where $\mathbb{1}_N$ is the $N \times N$ identity matrix. Eq. (28) and (29) require that

$$\mathbf{D}^{-1} = \begin{pmatrix} \mathbf{u} & -\mathbf{v} \\ -\mathbf{v} & \mathbf{u} \end{pmatrix}, \tag{30}$$

which enables us to write the diagonalization problem as an eigenvalue problem of the form

$$\begin{pmatrix} \mathbf{\Gamma} & -\mathbf{\Lambda} \\ \mathbf{\Lambda} & -\mathbf{\Gamma} \end{pmatrix} = \mathbf{D} \begin{pmatrix} \mathbf{\Delta}(\mathbf{q}) & 0 \\ 0 & -\mathbf{\Delta}(\mathbf{q}) \end{pmatrix} \mathbf{D}^{-1}, \tag{31}$$

which means that to find the spin-wave spectra contained within $\mathbf{\Delta}$ we must find the eigenvalues of the left hand side of Eq. (31).

6 Zero-energy spin-wave band

To see that the the spin–waves of the spin liquid RKKY interaction on kagome have a zero-energy flat band for all $\mathbf{q}$ we use the fact that the determinant of the matrix in Eq. (31) is the product of the eigenvalues and so the matrix must have zero determinant – and hence be non–invertible – if it has a zero eigenvalue. Since a zero determinant is unchanged by addition of rows and columns; subtracting the second row from the first and then subtracting the first column from the second leads to,

$$\begin{pmatrix} \mathbf{\Gamma} & -\mathbf{\Lambda} \\ \mathbf{\Lambda} & -\mathbf{\Gamma} \end{pmatrix} \to \begin{pmatrix} \mathbf{\Gamma}-\mathbf{\Lambda} & -\mathbf{\Lambda}-\mathbf{\Gamma} \\ \mathbf{\Lambda} & -\mathbf{\Gamma} \end{pmatrix} \to \begin{pmatrix} \mathbf{\Gamma}-\mathbf{\Lambda} & 0 \\ \mathbf{\Lambda} & -\mathbf{\Gamma}-\mathbf{\Lambda} \end{pmatrix}, \tag{32}$$

which is now in the form of a block triangular matrix. We can therefore write the determinant as $\Delta = \det\{\mathbf{\Gamma}-\mathbf{\Lambda}\}\det\{-\mathbf{\Gamma}-\mathbf{\Lambda}\}$. From the definitions of $\mathbf{\Gamma}$ and $\mathbf{\Lambda}$ the first block matrix in terms of the susceptibility reads

$$[\mathbf{\Gamma}]_{ab} - [\mathbf{\Lambda}]_{ab} = 2\delta_{ab}\sum_c \cos(\theta_a - \theta_c)\chi_{ac}(\mathbf{Q}_n) - 2\chi_{ab}(\mathbf{Q}_n + \mathbf{q}) = 2\lambda_{\min}\delta_{ab} - 2\chi_{ab}(\mathbf{Q}_n + \mathbf{q}), \tag{33}$$

where the $\lambda_{\min}$ in the final line follows from the choice of the spin orientations θ_i such that we achieve the mean field minimum energy. The matrix in Eq. (33) therefore has a zero-eigenvalue from the cancellation of the diagonal of the first term and the $\lambda_{\min}$ flat band of the susceptibility, $\chi^{ab}(\mathbf{Q}_n + \mathbf{q})$. From this it follows that $\det\{\mathbf{\Gamma}-\mathbf{\Lambda}\} = 0$ and hence the original Bogoliubov matrix of spin-wave excitations in Eq. (32) as has a zero-energy spectrum for all $\mathbf{q}$.

7 Flat linear order spin wave band from hard constraint on spin length

In order to understand the origin of the flat-band it is instructive to consider the full classical equation of motion of the spins such that we treat them as classical vectors $\mathbf{I}_{i,m}$ (where i labels the unit cell and m the sublattice). The dynamics of these spins are set by the slRKKY Hamiltonian and they have the (classical) equation of motion

$$\frac{d\mathbf{I}_{i,m}}{dt} = -2\mathbf{I}_{i,m} \times \sum_{j,n} \mathbf{I}_{j,n} \chi_{ij}^{mn}. \tag{34}$$

Which in momentum space reads:

$$\frac{d\mathbf{I}_{\mathbf{q},m}}{dt} = -2\sum_{\mathbf{k}} \mathbf{I}_{\mathbf{q}-\mathbf{k},m} \times \sum_{n} \mathbf{I}_{\mathbf{k},n} \chi^{mn}(\mathbf{k}). \tag{35}$$

If the classical nuclear spins are ordered then we can expand them about the mean field ordering direction such that $\mathbf{I}_{i,m} = \langle \mathbf{I}_{i,m} \rangle + \delta \mathbf{I}_{i,m}$ or in Fourier space $\mathbf{I}_{\mathbf{q},m} = \langle \mathbf{I}_{\mathbf{Q}_{\rm ord},m} \rangle \delta_{\mathbf{q},\mathbf{Q}_{\rm ord}} + \delta \mathbf{I}_{\mathbf{q},m}$, which at linear order in the expansion will be a fully classical equivalent to the Holstein-Primakoff expansion. Inserting this into Eq. (35) we obtain to linear order the estimate of the classical equation of motion for the spin in a unit cell

$$\frac{d\delta\mathbf{I}_{\mathbf{q},m}}{dt} = 2\sum_{n} \boldsymbol{R}(\theta_n)\delta_{nm}\left(\sum_{l}(\chi^{ln}(\mathbf{Q}_{\rm ord})\langle \mathbf{I}_{\mathbf{Q}_{\rm ord},l}\rangle - \chi^{mn}(\mathbf{Q}_{\rm ord}-\mathbf{q})\langle \mathbf{I}_{\mathbf{Q}_{\rm ord},n}\rangle)\boldsymbol{R}(\theta_m)\right) \times \delta\mathbf{I}_{\mathbf{q},m}, \tag{36}$$

where $\boldsymbol{R}(\theta_j)$ are defined in the main text. From this we see that the matrix governing the classical motion of the spins will also have a zero eigenvalue since the constant eigenvalue of the two matrices will cancel.

To understand why in a system where the energy minima result entirely from the constraint on spin length necessarily has a flat band at linear order (but can be dispersive at higher orders) in spin length, consider the hard constraint for classical spins in real space $\mathbf{I}_{i,m} \cdot \mathbf{I}_{i,m} = I$. This constraint can be written in terms of spins fluctuating around some equilibrium position $\langle I \rangle$ and requires $|\langle I \rangle + \delta I|^2 = I^2$. After expanding this reads $2\delta I \cdot \langle I \rangle + |\delta I|^2 = I^2 - |\langle I \rangle|^2 \geq 0$, where in the last line we obtain the inequality because the component of spin in the direction of position $|\langle I \rangle|$ must be smaller or the same size as I for a fixed spin length. Spin waves are transverse and so $2\delta I \cdot \langle I \rangle = 0$. We see therefore that for finite fluctuations the inequality can only be satisfied – and hence the hard constraint on spin length satisfied – by high orders in δI. As such only higher than linear order fluctuations can enforce the true constraint on spin length and, if the system solely achieves its energy minima because of this constraint, only these higher order terms will capture the increase in energy resulting from the ability of the spins to fluctuate. The above argument holds not only for regular periodic orders, but also for the multiple-$\boldsymbol{Q}$ states.

8 Effect of nuclear (Overhauser) field on the KHAF

As mentioned in the main text, after ordering of the nuclear spins there exists an Overhauser field of the nuclear moments on the spin liquid with the nuclear spins form an effective magnetic field. In principle this field could have a destabilizing effect on the spin liquid. Here we discuss why this is unlikely to impact our conclusions relating to nuclear orderings. Although such a back action may be of interest for a discussion of the KHAF spin-liquid state in real materials at very low temepratures, the focus of this work was on orderings of nuclear moments.

There are two effects from the reciprocal action that could qualitatively affect the physics of the nuclear moments: 1) a significant change in the susceptibility or 2) the loss of the spin liquid's dynamics.

1) As was shown in detail in the previous sections and main text, the coincidence of the symmetries of the lattice of the electron spins and nuclear spins allows only certain high symmetry positions within reciprocal space to minimise the slRKKY energy. The coincidence also means that any feedback between the two species of spins must maintain the symmetries of the susceptibility. Since the interactions leading to the spin liquid itself are constrained to the same symmetries both J and A act on the same scales. But since $A\langle I \rangle \ll J$ the effect from the Overhauser field is weak.

2) Other than the susceptibility, the other key consideration in the calculation of nuclear orders is that the spin liquid remains dynamic – at least down to temperatures at which the nuclear spins order. This consideration is in fact related to the question of the precise nature of the spin liquid, which for instance in the case of KHAF is still undecided despite extensive research over many years. However, as the slRKKY physics is dictated by its symmetries, only the still unanswered question whether or not the ground state of the $S = 1/2$ KAFM is gapped or gapless can be of importance for the effect of the Overhauser field. In addition the question has two facets, whether the Overhauser field plays a role in principle or whether the reciprocal action matters in practice at the relevant temperatures.

We address first the question in principle: For a gapped spin liquid, the Overhauser field could enforce or compete against the gap. But even a small gap such as $\Delta \sim 0.05 - 0.1J$[8,9] is still very large in comparison to the Overhauser field's back action

as $A\langle I\rangle \ll \Delta$. This makes the back action highly unlikely to destabilise the spin liquid. For a gapless spin liquid[10,11] the question is if the Overhauser field could freeze the dynamics by opening a gap itself. It is not possible to conclusively say whether the Overhauser field will open a gap, since even the nature of the kagome spin liquid without any back action is not fully resolved, a gap could open up that is capable of modifying the temperature scales discussed in the remainder of the manuscript. However, as argued in 1) even if a gap is opened up it is unlikely to freeze the dynamics of the spin liquid since the Overhauser field acts on the same underlying lattice as the kagome spin liquid so that J and A act with the same weight and, since $A\langle I\rangle \ll J$, the feedback is not likely to open a sizeable gap capable of destroying the spin liquid.

Practically, however, it is important only that the spin liquid remains dynamic at temperatures corresponding to those which the nuclear spins have already ordered, T_N, even if a small gap does open that causes a loss of the spin liquid dynamics. If the freezing temperature of the spin liquid is smaller than T_N, then any opening of a gap would have no significant impact on the nuclear order. As no freezing of the spin liquid has been observed in kagome systems down to $40-50$ mK[12,13] it is quite safe to assume that realistically sufficient dynamics can be maintained at the nuclear ordering temperatures required.

References

1. Yamaji, K. & Kondo, J. Thermodynamics of the two-dimensional ferromagnetic Heisenberg spin systems. *Phys. Lett. A* **45**, 317 (1973).
2. Bernhard, B. H., Canals, B. & Lacroix, C. Green's function approach to the magnetic properties of the kagomé antiferromagnet. *Phys. Rev. B* **66**, 104424, DOI: https://doi.org/10.1103/PhysRevB.66.104424 (2002).
3. Yu, W. & Feng, S. Spin-liquid state for two-dimensional Heisenberg antiferromagnets on a kagomé lattice. *Eur. Phys. J. B* **13**, 265 (2000).
4. Tyablikov, S. Retarded and advanced green functions in the theory of ferromagnetism. *Ukrain. Mat.* **11**, 287 (1959).
5. Garanin, D. & Canals, B. Classical spin liquid: Exact solution for the infinite-component antiferromagnetic model on the kagomé lattice. *Phys. Rev. B* **59**, 443 (1999).
6. Chalker, J., Holdsworth, P. & Shender, E. Hidden order in a frustrated system: Properties of the Heisenberg kagomé antiferromagnet. *Phys. Rev. Lett.* **68**, 855 (1992).
7. Holstein, T. & Primakoff, H. Field dependence of the intrinsic domain magnetization of a ferromagnet. *Phys. Rev.* **58**, 1098, DOI: https://doi.org/10.1103/PhysRev.58.1098 (1940).
8. Yan, S., Huse, D. A. & White, S. R. Spin-liquid ground state of the $S=1/2$ kagome Heisenberg antiferromagnet. *Science* **332**, 1173–1176, DOI: https://doi.org/10.1126/science.1201080 (2011).
9. Nishimoto, S., Shibata, N. & Hotta, C. Controlling frustrated liquids and solids with an applied field in a kagome Heisenberg antiferromagnet. *Nat. Comm.* **4**, 2287 (2013).
10. Sakai, T. & Nakano, H. Gapless spin excitations in the $S=1/2$ kagome-and triangular-lattice Heisenberg antiferromagnets. *Phys. B Condens. Matter* **536**, 85 (2018).
11. Liao, H. J. *et al.* Gapless spin-liquid ground state in the $S=1/2$ kagome antiferromagnet. *Phys. Rev. Lett.* **118**, 137202, DOI: https://doi.org/10.1103/PhysRevLett.118.137202 (2017).
12. Mendels, P. *et al.* Quantum magnetism in the paratacamite family: Towards an ideal kagome lattice. *Phys. Rev. Lett.* **98**, 077204, DOI: https://doi.org/10.1103/PhysRevLett.98.077204 (2007).
13. Clark, L. *et al.* Gapless spin liquid ground state in the S=1/2 vanadium oxyfluoride kagome antiferromagnet $[NH_4]_2[C_7H_{14}N][V_7O_6F_{18}]$. *Phys. Rev. Lett.* **110**, 207208, DOI: https://doi.org/10.1103/PhysRevLett.110.207208 (2013).
